# The Human Urate Transportosome: Evolutionary Dynamics, Molecular Mechanisms, and Pharmacogenetic Perspectives

**DOI:** 10.3390/medsci14060612

**Published:** 2026-09-28

**Authors:** Lilya U. Dzhemileva, Vladimir A. D’yakonov, Sergey N. Marshala, Elza Khusnutdinova, Andrey A. Deviatkin, German A. Shipulin

**Affiliations:** 1 N.D. Zelinsky Institute of Organic Chemistry, Russian Academy of Sciences, Leninsky Prospect 47, Moscow 119991, Russia; 2 Federal State Budgetary Institution “Centre for Strategic Planning and Management of Biomedical Health Risks” of the Federal Medical and Biological Agency, Pogodinskaya Str. 10/1, Moscow 119121, Russia; 3 JSC “Medsi Group of Companies”, Michurinsky Prospekt 56/1, Moscow 119192, Russia; marshala_2011@mail.ru; 4 Department of Medical Genetics and Fundamental Medicine, Bashkir State Medical University, Ufa 450008, Russia; 5 Department of Propaedeutics of Internal Diseases No. 2, Institute of Clinical Medicine, Pirogov Russian National Research Medical University, Moscow 117513, Russia

**Keywords:** hyperuricemia, gout, urate transportosome, ABCG2, SLC2A9, HLA-B*58:01, pharmacogenomics

## Abstract

**Background:** The hominoid loss of urate oxidase (uricase) represents a classic evolutionary trade-off, shifting uric acid (UA) from a metabolic waste product to a potent physiological modulator. In modern metabolic environments, however, this adaptation drives hyperuricemia and gout, transforming UA into a primary pathological substrate. **Objective:** This review aims to dissect the molecular architecture and biophysical networks of the renal and intestinal urate transportosome, delineate the dual intracellular/extracellular “urate paradox,” and synthesize genotype-based pharmacogenetic strategies to achieve personalized clinical management. **Mechanistic Insights:** During the Miocene epoch, inactivating pseudogenization of the *UOX* gene fixed a novel metabolic phenotype characterized by fructose-driven lipid deposition and enhanced antioxidant protection. Structurally, systemic urate homeostasis is strictly governed by a macromolecular interactome assembled by the four-domain scaffold protein PDZK1 on the epithelial apical membrane. Pathogenic gain-of-function variants in reabsorption facilitators (*SLC22A12*/URAT1, *SLC2A9*/GLUT9) or loss-of-function mutations in the efflux pump (*ABCG2*/BCRP) disrupt this delicate vector kinetics. Within the extracellular space, soluble urate acts as a critical hydrophilic radical scavenger. Paradoxically, upon URAT1/GLUT9-mediated internalization or intracellular supersaturation, intracellular urate triggers a pro-oxidant cascade mediated by NADPH oxidase (NOX4) activation and mitochondrial electron transport chain decoupling. This chronic cellular stress activates downstream p38 MAPK and NF-κB signaling pathways, driving localized endothelial injury and macrovascular inflammation, while crystalline monosodium urate (MSU) orchestrates NLRP3 inflammasome assembly in macrophages. **Pharmacogenetic Implications:** Striking ethno-geographic heterogeneity dictates immediate clinical stratification. The HLA-B*58:01 allele, an absolute molecular contraindication for allopurinol due to life-threatening severe cutaneous adverse reactions (SCARs), exhibits a critical genetic gradient in northern and eastern Eurasian populations, surging from under 1% in ethnic Caucasians to over 10% in indigenous populations of East/North Asian ancestry. Furthermore, structural defects in ABCG2 (such as the p.Q141K variant) alter the ATP-binding cassette domain, inducing standard allopurinol resistance and elevated statin exposure, which mandates a therapeutic pivot toward selective xanthine oxidase inhibitors (febuxostat) or precision uricosurics (benzbromarone, dotinurad) matched to the patient’s interactive network profile. **Conclusions:** Transitioning from generalized epidemiological guidelines to a comprehensive “transportosome genetic passport” is a fundamental prerequisite for predicting single-nucleotide polymorphism (SNP)-driven therapeutic responses and mitigating visceral complications.

## 1. Introduction

### Literature Search Strategy and Selection Criteria

To ensure a comprehensive, rigorous, and reproducible synthesis of the evolutionary, structural, and pharmacogenetic architecture of hyperuricemia and gout, a systematic literature search was executed across major international biomedical databases, including PubMed/MEDLINE, Scopus, and the Web of Science Core Collection up to 2026. The search string architecture utilized combinations of controlled vocabulary (Medical Subject Headings [MeSH] terms) and high-affinity text keywords. The primary search strategy incorporated the following Boolean operators and terms: (“hyperuricemia” OR “gout”) AND (“urate transportosome” OR “urate oxidase” OR “uricase” OR “UOX gene pseudogenization”) AND (“ABCG2” OR “BCRP” OR “SLC2A9” OR “GLUT9” OR “SLC22A12” OR “URAT1” OR “PDZK1 scaffold”) AND (“HLA-B*58:01” OR “pharmacogenomics” OR “severe cutaneous adverse reactions” OR “SCAR”) AND (“thrifty genotype hypothesis” OR “monogenic metabolic syndromes”). Manuscripts retrieved from the systematic search were dynamically screened based on rigorous expert consensus criteria. The inclusion criteria were strictly restricted to peer-reviewed, high-impact studies encompassing: large-scale genome-wide association studies (GWASs), fine-mapping consortia, and meta-analyses exploring reference single-nucleotide polymorphisms (SNPs) within renal and intestinal transporter loci; comparative evolutionary genetics and phylogenetic medicine studies investigating anthropoid ancestral UOX gene inactivation; prospective randomized controlled trials, effectiveness studies, and pharmacogenomic screening assays evaluating therapeutic responses, cellular resistance, and substrate toxicity profiles of urate-lowering agents (e.g., allopurinol, febuxostat, benzbromarone, dotinurad, lesinurad); comprehensive clinical and molecular studies defining geographic heterogeneity, population genetics, and multi-system metabolic comorbidities in predefined ethnic cohorts. Conversely, publications were excluded if they comprised non-peer-reviewed preprints (unless serving as definitive historical benchmarks), non-systematic consensus expert opinions, generalized textbook chapters, or descriptive clinical protocols lacking rigorous molecular, cellular, or structural biophysical validation criteria. Literature screening, identification, data extraction, and subsequent synthesis were performed in strict compliance with the Preferred Reporting Items for Systematic Reviews and Meta-Analyses (PRISMA) guidelines, transitioning the raw bibliographical data into a mechanistic structural network.

## 2. Metabolism and Excretion Pathways of Uric Acid in Humans

The homeostatic regulation of purine metabolism represents a critical evolutionary and metabolic axis in hominoid physiology. In humans and higher primates, the systemic concentration of circulating urate is determined by a delicate kinetic balance between endogenous production, dietary purine ingestion, and structural elimination pathways [1,2]. While approximately one-third of the human purine pool originates from exogenous dietary sources, the remaining two-thirds are synthesized endogenously via cell turnover and de novo purine biosynthetic pathways [3,4,5]. Under physiological conditions, uric acid (UA) serves as the definitive end product of this metabolic cascade. To date, there is no universally accepted biochemical definition of hyperuricemia, and contemporary clinical practice recognizes distinct conceptual frameworks [1,2,3]. For purposes relating to pathogenic urate crystal deposition, a physicochemical definition of hyperuricemia corresponds to serum uric acid (S-UA) concentrations exceeding approximately 420 µmol/L (7.0 mg/dL), which represents the precise solubility limit of monosodium urate in human body fluids at physiological temperature. Conversely, many experts advocate for an alternative, practical, and clinically relevant definition of hyperuricemia set at an S-UA threshold exceeding 360 µmol/L (6.0 mg/dL) [3,6]. This lower value integrates the estimated lifelong risk for long-term clinical consequences of hyperuricemia with the universally recommended target range of <360 µmol/L required for successful urate-lowering therapy in gout [2,6]. Furthermore, epidemiological and diagnostic criteria vary greatly regarding sex-specific and age-dependent distinctions, with established hyperuricemia cutoffs frequently defined as >420 µmol/L for adult men and >360 µmol/L for adult women and pediatric patients under 15 years of age [1,3]. Recent global conjoined analyses from 2019 to 2021 indicate that the global burden of gout has reached approximately 57 million cases, with a standardized incidence rate of 9.4 million new cases annually, translating to an immediate loss of 1.75 million disability-adjusted life years (DALYs) [4,5,6].

From a pathogenetic and mechanistic perspective, systemic hyperuricemia is strictly heterogeneous. Although uric acid hyperproduction can be driven by intensive cytolysis, high cellular turnover, or clinical pathologies such as acute hemolysis, rhabdomyolysis, and tumor lysis syndrome, these overproduction phenotypes constitute only a fraction of clinical presentations. In the vast majority of patients (approximately 90%), the dominant pathogenetic driver is a significant reduction in the excretory capacity of the renal and intestinal epithelium [7]. Human uric acid elimination utilizes a dual pathway comprising renal clearance (approximating 70% of total urate removal) and extrarenal intestinal secretion (accounting for the remaining 30%). Crucially, the ATP-binding cassette transporter ABCG2 serves as a shared high-capacity master valve across both pathways, gating apical urate efflux in both the renal proximal tubule epithelium and intestinal enterocytes [8].

Despite free glomerular filtration in the nephron, up to 90% of filtered urate undergoes intensive, energy-dependent reabsorption within the proximal convoluted tubules. This massive vectorial transport is not mediated by isolated, independent membrane channels, but is governed by a highly integrated macromolecular network termed the urate transportosome. The key structural components regulating this proximal tubular antiport and facilitated diffusion kinetics are the URAT1 and the basolateral efflux facilitator GLUT9, working in tight spatial orchestration with active secretory pumps such as ABCG2 [8]. The pathogenetic transition from subclinical hyperuricemia to symptomatic gouty arthritis or urate nephrolithiasis occurs when circulating concentrations exceed the physiological solubility threshold of monosodium urate (MSU) in aqueous solution (≈380 μmol/L), triggering a physical phase transition and tissue crystal deposition [9,10].

A definitive feature of mammalian purine metabolism is the structural transformation of the purine degradation cascade. In most non-primate mammals, xanthine oxidase catalyzes the sequential oxidation of hypoxanthine to xanthine, and xanthine to uric acid, which is then rapidly targeted for enzymatic uricolysis by hepatic urate oxidase (uricase) [11]. This evolutionarily conserved enzyme mediates the conversion of poorly soluble uric acid into highly hydrophilic allantoin, which possesses a high saturation threshold in aqueous solutions and is freely excreted by the kidneys without substantial reabsorption [12,13]. However, the human lineage, alongside specific higher primates (chimpanzees, gorillas, orangutans, and gibbons), lacks a functional uricase enzyme due to nonsense and splice-site mutations that pseudogenized the UOX gene [14,15]. Consequently, free uric acid serves as the ultimate, non-degradable metabolite of purine breakdown in humans, exhibiting pronounced hydrophobicity and a remarkably low solubility limit in systemic circulation [12]. Notably, while humans lack a functional, genetically encoded hepatic uricase enzyme, alternative non-canonical pathways for urate degradation still operate within the systemic and cellular matrices. Uric acid can be systematically oxidized to allantoin via non-enzymatic pathways mediated directly by reactive oxygen species (ROS) or reactive nitrogen species under conditions of systemic oxidative stress [6]. Furthermore, during intense neutrophilic inflammation—such as acute gouty flares—circulating urate serves as a substrate for enzymatic oxidation mediated by leukocyte-derived myeloperoxidase, presenting a vital, compensatory chemical buffer against extreme local purine supersaturation [6]. While a prolonged asymptomatic course is observed in approximately 85–90% of individuals with elevated urate levels, persistent extracellular supersaturation serves as an obligate pathogenetic driver for micro-crystal precipitation, culminating in acute gouty arthritis or urate nephrolithiasis [9,16]. While classical epidemiological data robustly link chronic hyperuricemia with an array of metabolic and cardiovascular comorbidities, critical insights from recent Mendelian randomization (MR) studies necessitate a strict delineation between mere statistical association and direct pathogenetic causation [17]. True causal relationships have been firmly established for gouty arthritis, where Mendelian randomization confirms a direct, genetically driven etiology mediated by monosodium urate crystal deposition [16,17]. Similarly, supportive causal evidence exists connecting sustained hyperuricemia with arterial hypertension and elevated blood pressure [17,18]. However, for coronary heart disease (CHD), the data remain highly heterogeneous, showing supportive causal links only in specific stratified sub-analyses [19]. Conversely, in the context of chronic kidney disease (CKD) and estimated glomerular filtration rate (eGFR) decline, MR analyses frequently yield weak or even negative causal evidence, suggesting that the observed clinical correlations may stem from reverse causality, wherein impaired renal clearance drives urate retention rather than vice versa [17,20]. Furthermore, the hypothesized causal involvement of serum urate in the pathogenesis of type 2 diabetes, metabolic syndrome, and neurodegenerative disorders remains considerably less convincing than previously suggested by observational cohorts; hence, epidemiological assertions must be moderated to reflect these non-causal genetic insights [1]. Bidirectional pathogenetic links establish a vicious cycle: reduced glomerular filtration and altered fractional excretion drive systemic urate retention, while persistent hyperuricemia actively accelerates arteriolosclerosis, glomerulosclerosis, and tubulointerstitial kidney fibrosis [20]. Concurrently, large-scale studies verify comorbid cardiovascular pathologies—including arterial hypertension, advanced atherosclerosis, chronic heart failure, and stroke —in approximately 41% of patients diagnosed with gout [21,22,23,24,25,26,27,28,29]. This systemic vascular and metabolic pathogenicity is governed by the structural dichotomy known as the “urate paradox” [20]. Within the extracellular environment, soluble urate anion acts as a primary hydrophilic antioxidant, contributing up to 60% of total human plasma antioxidant capacity by chelating transition metals and directly scavenging reactive oxygen species (ROS) and peroxynitrites [19,20]. However, this protective matrix shifts dramatically upon molecular internalization. When urate anions are intensively transported into vascular endothelial cells and intracellular cytosolic matrices via specific transportosome channels (such as URAT1 and basolateral GLUT9 facilitation), they trigger a profound pro-oxidant cascade [10]. At the cellular level, elevated intracellular urate induces structural assembly and hyperactivation of NADPH oxidase 4 (NOX4), generating an immediate surge of intracellular superoxide radicals. This targeted oxidative stress provokes direct mitochondrial dysfunction by decoupling the mitochondrial electron transport chain and depleting intracellular adenosine triphosphate (ATP) pools. The resulting localized redox imbalance activates downstream mitogen-activated protein kinase (p38 MAPK) and nuclear factor kappa B (NF-κB) signaling networks. The activation of these transcription factors orchestrates the transcription of pro-inflammatory cytokines, suppresses endothelial nitric oxide synthase (eNOS) phosphorylation, and halts nitric oxide generation. This molecular cascade establishes sustained endothelial injury, structural vascular remodeling, and electrical instability within myocardial tissues—empirically linking genetic transportosome variants to accelerated atherosclerosis, coronary lesions, and atrial fibrillation [18] (Figure 1).

The poorly understood genetic heterogeneity of hyperuricemia, combined with the complex phenotypic and genetic polymorphism of the disease, results in significant diversity within its genetic and, particularly, pharmacogenetic profiles. These factors preclude the establishment of a universal ‘gold standard’ for treatment without considering regional genetic characteristics. This underscores the urgency of investigating the genetic diversity underlying phenotypic manifestations, the necessity of developing ethno-specific approaches to gout management, and the diagnostic significance of identifying asymptomatic forms to implement early preventive measures.

## 3. Genetic Features of Hyperuricemia and Evolutionary Aspects of the UOX Locus

The phylogenetic lineage of higher primates and humans is characterized by a profound metabolic shift driven by the systematic functional silencing of the *UOX* gene, which encodes the peroxisomal enzyme urate oxidase (uricase) [13,14]. In the vast majority of terrestrial mammals, uricase maintains systemic urate levels within a strict physiological window by catalyzing the hydrolytic oxidation of poorly soluble uric acid into highly soluble allantoin [11,12]. However, during the Miocene epoch, approximately 15 to 20 million years ago, ancestral hominoids underwent a series of independent, sequential mutations within the *UOX* locus [14].

Molecular clock analyses and fine-mapping of the *UOX* pseudogene reveal that this evolutionary loss occurred through a multistep process involving a critical nonsense mutation, p.Arg33Ter (exon 2), in the common ancestor of humans and great apes (including orangutans), whereas distinct, independent inactivating mutations were fixed separately within the gibbon (Hylobatidae) lineage [14,15]. This evolutionary event induced irreversible pseudogenization of the *UOX* gene, resulting in a complete absence of functional uricase expression in human hepatic peroxisomes and causing baseline serum urate concentrations to surge up to tenfold compared to non-primate mammalian species [15]. Crucially, this evolutionary trajectory was not an isolated UOX-centered phenomenon but involved an intricate molecular coevolution with key transportosome entities, specifically URAT1 and the newly neofunctionalized primate urate transporter SLC52A1. Experimental ancestral-reconstruction studies demonstrate that during primate evolution, the functional selection for elevated systemic urate levels was driven by coordinated changes across multiple loci [30]. As hepatic uricase activity progressively declined due to accumulating mutations, the kinetic properties of URAT1 adapted simultaneously; its affinity for urate significantly increased, while its maximum transport capacity decreased. This coordinated shift optimized the retention of filtered urate under fluctuating environmental pressures. Concurrently, the neofunctionalization of SLC52A1 emerged as a critical adaptive response, enabling active intestinal urate secretion to mitigate catastrophic systemic overload [31]. This multigenic adaptation highlights a complex evolutionary trade-off, where alternative hemodynamic and metabolic advantages were prioritized at the expense of baseline purine clearance stability.

From an evolutionary medicine perspective, the fixations of these disruptive mutations within the hominoid genome are fundamentally described by James Neel’s “thrifty genotype hypothesis” [25]. During the mid-Miocene cooling transition, early hominoids faced severe dietary restriction, environmental disruption, and localized famine [13,14,26]. In this ecological context, the loss of uricase provided a profound survival advantage. Soluble intracellular urate serves as a key metabolic amplifier that intensifies fructose-mediated lipogenesis and accelerates de novo fatty acid synthesis within hepatocytes [25].

By driving local mitochondrial oxidative stress via the accumulation of intra-mitochondrial urate, elevated levels suppress enoyl-CoA hydratase 1, thereby impeding beta-oxidation while simultaneously promoting triglyceride accumulation [32,33]. This targeted energetic shift allowed hominoids to efficiently convert scarce dietary fructose from seasonal fruits into essential fat reserves, mitigating starvation risks and enabling expansion across altered geographical niches [14,33,34]. Furthermore, the evolutionary maintenance of high urate levels has been linked to the maintenance of arterial blood pressure under conditions of low dietary sodium intake, and it provided a critical neuroprotective matrix that supported the expansion of cognitive capacities and cortical development in higher primates [28,34].

However, in modern dietary and metabolic environments characterized by an abundance of purine-rich foods, high-fructose corn syrup, and extended lifespans, this once-beneficial genetic adaptation has transformed into a primary pathological driver of metabolic syndrome, type 2 diabetes, and cardiovascular injury [27,35]. Paradoxically, at the interface of innate immunity and molecular pathophysiology, intracellular urate acts as a potent endogenous Danger-Associated Molecular Pattern (DAMP), or “danger signal” [33].

Upon physical cellular disruption, or when intracellular urate supersaturation triggers the precipitation of microscopic monosodium urate (MSU) clusters within the cytoplasmic matrix, the molecule directly triggers the structural assembly of the NLRP3 inflammasome complex [36]. Structurally, intracellular urate concentration fluctuations activate the cell-surface purinergic receptors and modulate the heat shock protein network, leading to the recruitment of apoptosis-associated speck-like protein containing a CARD (ASC) and the subsequent activation of caspase-1 [36]. This enzymatic cascade induces the proteolytic processing and secretion of highly pro-inflammatory cytokines, specifically interleukin-1 beta (IL-1β) and interleukin-18 (IL-18) [37].

Furthermore, this intracellular DAMP activity orchestrates the immediate phenotypic maturation of dendritic cells, upregulates the expression of co-stimulatory molecules (such as CD80, CD86, and CD40), and promotes robust antigen-presenting functionality [38]. This sustained activation of localized endothelial matrices and tissue-resident macrophages initiates a self-amplifying inflammatory loop, directly linking the ancient Miocene pseudogenization of *UOX* to modern systemic vasculopathy, chronic low-grade vascular inflammation, and progressive atherogenesis [33].

## 4. The Molecular Architecture of the Urate Transportosome

The homeostatic maintenance of systemic urate concentrations in humans is strictly dictated by the coordinated transport kinetics of a macromolecular assembly localized within the polarized epithelial cells of the renal proximal tubule and the intestinal mucosa, termed the urate transportosome [39]. Rather than operating as isolated membrane channels, these transport proteins form a spatially integrated interactive network on the apical and basolateral membranes. This structural compartmentalization allows the cell to execute rapid, vector-specific shifts between reabsorption and secretion in response to fluctuating metabolic demands [39]. The foundational framework of this multi-protein transport machine is physically organized by the sub-membrane scaffold protein PDZK1 (encoded by the PDZK1 gene at locus 1q21.1) [40,41]. PDZK1 is a multivalent adaptor containing four highly conserved PSD-95/Discs Large/Zonula Occludens-1 (PDZ) domains, each exhibiting high stereospecific affinity for the specific C-terminal motifs of distinct transport proteins [42]. The hierarchical organization of the transportosome relies on physical cross-linking mediated by these domains: the PDZ1 domain of PDZK1 binds directly to the COOH-terminus of the primary apical urate-anion exchanger URAT1 (SLC22A12), anchoring it in close proximity to other co-transporters [42]. Recent breakthroughs in cryogenic electron microscopy (cryo-EM) have dramatically advanced our understanding of the molecular architecture and transport dynamics of human URAT1 (encoded by SLC22A12). Specifically, recent structural determinations have resolved human URAT1 in its native state, urate-bound state, and in complex with various anti-gout inhibitors, providing an unprecedented view of its substrate selection and gating mechanisms [43,44,45]. The transport cycle of URAT1 is governed by distinct conformational transitions between outward-open, occluded, and inward-open states. Binding of the urate substrate within the central hydrophilic cavity induces micro-conformational shifts that facilitate electroneutral antiport exchange. Furthermore, high-resolution structural mapping has elucidated the molecular basis of URAT1 inhibition by conventional and novel anti-gout drugs [46]. Uricosuric agents (such as benzbromarone and dotinurad) directly access the central cavity, locking the transporter in an inactive outward-facing conformation through high-affinity hydrophobic interactions and steric occlusion, thereby effectively blocking luminal urate reabsorption [44]. Through its adjacent PDZ domains, PDZK1 physically recruits the sodium-coupled monocarboxylate transporters SMCT1 (SLC5A8) and SMCT2 (SLC5A12) [42]. This spatial coupling establishes a highly localized functional antiport microenvironment. SMCT1/2 utilize the transmembrane sodium gradient to drive the influx of intracellular monocarboxylates (such as lactate, nicotinate, and pyrazinoate), which are then immediately utilized by URAT1 as exchange substrates to power the electroneutral reabsorption of luminal urate from the primary urine into the cytosol [42,47]. Concurrently, PDZK1 coordinates the apical localization of organic anion transporter 4 (OAT4, SLC22A11) and the urate-modulated secretory channels NPT1 (SLC17A1) and NPT4 (SLC17A3), facilitating a highly efficient, localized substrate-channeling mechanism [44]. Single-nucleotide polymorphisms (SNPs) within the PDZK1 locus disrupt the structural affinity of these PDZ domains for their target peptides, leading to the diffuse mistargeting of transporters across the plasma membrane. This structural uncoupling impairs overall transport kinetics and clearance efficiency, manifesting clinically as persistent hyperuricemia and elevated gout susceptibility even in the presence of normal transporter expression levels [48].The definitive vector for transcellular urate reabsorption requires a two-step sequential transport cascade across the polarized epitheliocyte [44]. Following initial URAT1-mediated influx across the apical membrane, the intracellular urate pool is targeted for basolateral exit into the interstitial space and systemic circulation via the high-capacity glucose/fructose/urate transporter GLUT9 (encoded by the SLC2A9 gene at locus 4p16.1) [48,49]. Genome-wide association studies (GWASs) consistently validate SLC2A9 and SLC22A12 as the most robust genetic determinants of serum urate variance across global populations, demonstrating strong reproducibility across diverse ethnic cohorts [49,50]. Alternative splicing of the 5′ end of the SLC2A9 gene yields two structurally distinct isoforms—GLUT9S (short) and GLUT9L (long)—which exhibit strict differential subcellular localization [51,52,53]. GLUT9S is targeted predominantly to the apical membrane, where it may modulate initial urate influx alongside URAT1, whereas GLUT9L is localized exclusively to the basolateral membrane, providing the primary thermodynamic exit pathway driven by the transmembrane electrochemical gradient [54,55,56]. Gain-of-function variants within the SLC2A9 locus enhance mRNA stability or membrane expression density, inducing a synergistic hyperfunction of both isoforms [57]. This pathogenetic acceleration of vectorial reabsorption drives a critical reduction in the fractional excretion of uric acid (FEUA), establishing a distinct “underexcretor” renal phenotype and driving stable, long-term systemic hyperuricemia [58]. The crucial role of SLC22A12 and SLC2A9 as primary urate reuptake facilitators is further underscored by their involvement in hereditary renal hypouricemia (OMIM #220150—RHUC1; OMIM #612076—RHUC2), a condition documented across geographically and ethnically diverse populations [58,59]. Loss-of-function variants in SLC22A12, occurring in homozygous or compound heterozygous states, serve as the primary genetic etiology of RHUC, accounting for over 90% of documented cases and demonstrating profound population specificity [59,60]. According to recent HGMD Professional data, 81 distinct variants have been described in the SLC22A12 gene—encompassing 66 missense/nonsense, two splicing, three regulatory, two small insertions, six small deletions, one gross deletion, and one complex rearrangement—of which 52 are functionally characterized as definitive causes of renal hypouricemia type 1 [61]. The highest global prevalence of RHUC type 1 is clustered within Japanese and Korean populations, corresponding to an allelic frequency of 2.3% for the founder nonsense variant rs121907892 (p.W258X), whereas this specific allele remains exceptionally rare or entirely absent in African, American, South Asian, and European cohorts [59]. Notably, a high frequency of distinct, population-specific RHUC1 variants has been unveiled among European Roma cohorts (including Slovak, Czech, Spanish, and Macedonian subcohorts), where the missense variant rs200104135 (p.T467M) is present in 5.6% of individuals, and the deletion variant p.L415_G417 occurs in 1.9% [60]. Conversely, renal hypouricemia type 2 (RHUC2), caused by defects in the SLC2A9 gene, is rarer, with approximately 30 cases documented globally across diverse ethnicities (including Japanese, Chinese, Ashkenazi Jewish, Anglo-Saxon, Greek, Slovak, and Czech). HGMD Professional records delineate 51 variants in the SLC2A9 gene (comprising 35 missense/nonsense, four splicing, four regulatory, two small insertions, and two gross deletions), with 32 variants verified to cause RHUC2. Interestingly, RHUC2 exhibits greater clinical and biophysical variability than RHUC1 [62]. While heterozygous mutations in SLC2A9 yield serum uric acid profiles comparable to those of compound heterozygous or homozygous SLC22A12 mutations, homozygous or compound heterozygous RHUC2 patients present with extreme phenotypes, where serum uric acid concentrations approach absolute zero and fractional renal excretion markedly exceeds 100% [63,64]. Active urate secretion into the tubular and intestinal lumen counterbalances this reabsorption machinery and is primarily mediated by the efflux pump ABCG2 (BCRP, encoded by ABCG2 at locus 4q22.1) [17,65]. ABCG2 is a high-affinity, homodimeric ATP-binding cassette (ABC) efflux transporter localized on the apical membranes of both the renal proximal tubule epithelium and, to a significantly greater extent, the intestinal enterocytes [66,67]. Consequently, genetic defects in ABCG2 disrupt a dual-phased physiological axis: they compromise extrarenal intestinal secretion while simultaneously impairing active renal tubular secretion. This synchronized loss of function reduces overall extrarenal and renal transport kinetics by approximately 50%, generating a cumulative ‘underexcretor’ phenotype and accelerating the saturation of adjacent transportosome reabsorption channels [67,68]. The classic p.W258X and p.R90H loss-of-function variants in SLC22A12 represent a profound example of the founder effect in East Asian populations, being virtually absent in Caucasian cohorts [68]. Similarly, distinct geographic and ethnic gradients shape the distribution of SLC22A12 polymorphisms in isolated genetic isolates such as Iraqi Jews and the Roma population [68,69]. The precise structural mapping of these transportosome networks underscores that genetic variations altering protein-protein scaffolds or membrane channel thermodynamics constitute the primary molecular substrate dictating individual urate clearance profiles, population-specific disease incidence, and distinct pharmacogenetic phenotypes (Figure 2) [59].

In addition to the primary reabsorption and efflux channels, a comprehensive evaluation of the transportosome interactome necessitates the inclusion of organic anion transporter 1 (OAT1, encoded by SLC22A6) and organic anion transporter 3 (OAT3, encoded by SLC22A8). Recent comprehensive sequencing of diverse hyperuricemia and gout cohorts has enabled the functional characterization of several rare, non-synonymous variants within the SLC22A6 and SLC22A8 loci. These rare genetic mutations significantly impair baseline transport kinetics, protein trafficking, or substrate-binding affinities, thereby altering the net secretory capacity of the basolateral membrane and contributing to individual susceptibility to uric acid underexcretion.

## 5. Pharmacogenetics of Urate Transporters and Population Heterogeneity

The pronounced genetic polymorphism inherent within the human urate transportosome directly translates into extensive inter-individual and inter-ethnic variability regarding therapeutic efficacy, drug resistance, and xenobiotic toxicity profiles [60,61,62]. Mapping these underlying molecular mechanisms enables a precise transition from empiric, population-wide therapeutic strategies to targeted, pharmacogenetically guided interventions [62].

A baseline paradigm of precision uricosuric management involves the single-nucleotide polymorphism rs3825018 within the *SLC22A12* (URAT1) locus [50,70]. The rs3825018 G risk allele serves as a prominent genetic marker for hyperuricemia susceptibility, potentially driving altered transportosome efficiency through expression modulation (eQTL) rather than a classic structural gain-of-function mechanism [71]. Pharmacological blockade using benzbromarone—a direct, selective inhibitor of URAT1—specifically targets this hyperfunctional state, replicating the hereditary hypouricemia phenotype and restoring purine clearance [71]. Consequently, carriers of the G allele (harboring G/G and G/A genotypes) demonstrate superior responsiveness and a more pronounced delta in serum urate reduction compared to wild-type individuals [71,72].

Conversely, in patients carrying the inactivating loss-of-function variant p.R90H (rs121907896) or p.W258X (rs121907892), the administration of benzbromarone or other selective URAT1 inhibitors (e.g., lesinurad, dotinurad) is highly futile and potentially hazardous [71,72]. Because these specific transport channels are already genetically silenced, prescribing URAT1 inhibitors fails to yield any additional therapeutic benefit; these patients inherently exhibit baseline massive hyperuricosuria and are at a constant risk of exercise-induced AKI, regardless of uricosuric therapy [71,72].

Parallel pharmacodynamic interactions govern the *SLC2A9* (GLUT9) basolateral facilitation pathway [73,74,75]. Regulatory and intronic variants, including rs6855911, rs1014290, rs3775948, rs11722228, and rs6449213, dominate urate variance in European and East Asian cohorts [76,77]. Although these intronic alterations do not modify the primary amino acid sequence of the transporter, they reside in strong linkage disequilibrium (LD) with key promoters and enhancer regions, dictating the transcription rate, mRNA stability, and ultimate expression density of GLUT9 on the basolateral membranes [73,74,75,76,77,78,79]. Gain-of-function alleles (such as the rs6855911 G risk allele) generate an overexpressed transporter matrix that actively counteracts the therapeutic effects of conventional xanthine oxidase inhibitors, frequently resulting in allopurinol monotherapy failure and necessitating combination regimens [80,81,82,83,84,85].

Intriguingly, carriers of these *SLC2A9* risk variants exhibit a significantly potentiated uricosuric response during treatment with losartan [84]. Losartan modulates urate kinetics specifically via the inhibition of the apical transporter URAT1, without blocking basolateral OAT1/OAT3 secretion, positioning this agent as an ideal, personalized drug of choice for patients presenting with comorbid arterial hypertension and gout [84]. In a regional East European cohort study, the *SLC2A9* polymorphism rs734553 displays pronounced pleiotropic pathogenicity [82]. Beyond driving systemic hyperuricemia, carriage of the rs734553 C risk allele correlates with a 22% increase in the risk of atrial fibrillation (AF) and provokes stenotic coronary artery lesions 5–7 years earlier than in control cohorts [82]. This accelerated atherogenesis and electrical instability are driven by long-term systemic extracellular supersaturation and subsequent intracellular internalization via localized endothelial anion channels, accentuating cell-stress pathways [82,86,87,88].

The multixenobiotic efflux pump ABCG2 represents a critical node linking biophysical structural changes to multi-drug cumulative toxicity [86]. To date, approximately 50 non-synonymous variants have been identified within the *ABCG2* locus, many of which exhibit marked ethnic differences in allele frequency [87]. In sharp contrast to this high genomic variability, only two single-nucleotide polymorphisms—c.34G>A (p.V12M, rs2231137) and c.421C>A (p.Q141K, rs2231142)—are consistently recognized as common variants across most global ethnic groups [87,89]. The common dysfunctional variant rs2231142 (p.Q141K) fundamentally disrupts the nucleotide-binding domain (NBD) of the transporter, leading to protein instability and an approximate 50% reduction in urate efflux capacity [89,90,91]. Crucially, the population-attributable risk of ABCG2 dysfunction for hyperuricemia has been estimated at a striking 29.2%, an epidemiological impact that significantly exceeds that of conventional environmental risk factors such as a BMI ≥ 25.0 (18.7%), heavy alcohol consumption (15.4%), and age ≥ 60 years (5.74%). Clinically, the p.Q141K variant, along with the truncating nonsense variant rs72552713 (p.Q126X)—which is heavily enriched in East Asian and Japanese populations—shows an exceptionally strong association with an increased risk of hyperuricemia and familial, pediatric-onset gout (odds ratio > 3), often correlating with earlier disease onset, familial aggregation, and a trend toward lower BMI and C-reactive protein levels despite higher glomerular filtration rates [88,92,93]. Conversely, meta-analyses suggest a strong protective effect of the p.V12M variant against gout development (*p* < 0.0001); however, because function-altering cellular models in human embryonic kidney cells show no direct impact of p.V12M on baseline urate transport kinetics, this apparent protection is most likely attributable to linkage disequilibrium with other protective non-coding variants rather than a direct functional asset. In addition to these common polymorphisms, approximately 20 rare or ultrarare *ABCG2* variants (e.g., p.M131I, p.R236X, p.I242T, p.T434M) have been functionally characterized as severe loss-of-function defects that cause up to a complete loss of wild-type activity, making systematic *ABCG2* genotyping highly beneficial for patients presenting with early-onset or familial hyperuricemia [90,92]. The non-synonymous single-nucleotide polymorphism rs2231142 (p.Q141K) induces a missense substitution that fundamentally alters the tertiary structure of the ABCG2 monomer [84]. Biophysically, this substitution disrupts the ATP-binding cassette domain (nucleotide-binding domain, NBD), resulting in structural instability, defective protein folding, and an approximate 50% reduction in urate efflux capability [90,91,94,95,96,97,98,99,100,101]. Furthermore, misfolded p.Q141K proteins are rapidly recognized by the quality-control machinery of the endoplasmic reticulum, triggering intensive ubiquitination and premature proteasomal degradation [90]. This structural deficit drastically impairs extrarenal intestinal urate clearance, precipitating early-onset gout (manifesting before age 40) and severe pharmacoresistance to standard allopurinol titration (300 mg/day) [92,102].

Crucially, because ABCG2 serves as a shared transport pathway for numerous xenobiotics, the p.Q141K variant drives critical systemic drug–drug interactions [103,104,105]. In carriers of this risk allele, the hepatic and intestinal clearance of HMG-CoA reductase inhibitors—specifically rosuvastatin and atorvastatin—is profoundly compromised, increasing systemic plasma exposure by 1.5–2 times and exponentially elevating the clinical risk of statin-induced myalgia, muscle wasting, and rhabdomyolysis [106]. The rare p.Q126X (rs72552713) nonsense mutation, highly prevalent in Southeast Asian nations but virtually absent in Caucasians, introduces a premature stop codon that eliminates the functional protein matrix entirely, causing absolute cumulative toxicity for any ABCG2 substrate [91,92,98,99,100,101,102,103,104,105,106,107,108]. Conversely, the rs2231137 (p.V12M) variant exerts a protective effect, stabilizing membrane thermodynamics and reducing gout susceptibility in Asian cohorts [100,101].

Finally, the phenotypic manifestation of these genetic risk loci is strictly modulated by sexual dimorphism and hormonal axes [109]. Estrogens act as a potent physiological mask against genetic predisposition in premenopausal women by directly suppressing the transcriptional expression and apical/basolateral density of URAT1 and GLUT9 [110,111]. Consequently, *SLC2A9* variants exhibit a sex-specific effect size, demonstrating an exponential pathogenetic correlation predominantly in the female population [111]. The decline of estrogenic protection during postmenopause leads to the rapid decompensation of these hidden excretory defects [112]. In postmenopausal female carriers of the ABCG2 p.Q141K or *SLC2A9* risk alleles, urate levels experience an exponential surge, frequently manifesting as rapid-onset gouty arthritis complicated by secondary pharmacoresistance to conventional treatments, which mandates aggressive combination therapies or the precise evaluation of hormone replacement therapy (HRT) interactions [93].

## 6. Immune-Genetic Profiling: The HLA-B*58:01 Axis and Severe Cutaneous Adverse Reactions

The clinical implementation of urate-lowering therapies (ULT) requires careful management due to systemic immunogenetic liabilities driven by major histocompatibility complex (MHC) class I polymorphisms [113,114]. The single-nucleotide polymorphism defining the HLA-B*58:01 allele (localized on the short arm of chromosome 6 at locus 6p21.33) serves as an absolute, highly penetrant molecular predictor for the development of allopurinol-induced SCARs [113,115]. This group of life-threatening hypersensitivity syndromes encompasses Stevens–Johnson syndrome (SJS), toxic epidermal necrolysis (TEN), and Drug Reaction with Eosinophilia and Systemic Symptoms (DRESS) syndrome, all of which exhibit high mortality rates and severe systemic visceral complications [114].

At the molecular and immunogenetic interface, allopurinol-induced severe cutaneous adverse reactions (SCARs) are driven by a classic, T-cell-mediated delayed-type hypersensitivity response (Type IV) [116]. According to the established pharmacological interaction (p-i) and hapten concepts, oxypurinol—the primary, long-lived active metabolite of allopurinol—exhibits a high stereospecific affinity for the antigen-binding cleft of the structural HLA-B*58:01 proteins on antigen-presenting cells and keratinocytes without requiring prior intracellular processing. This direct non-covalent binding alters the surface charge profile of the major histocompatibility complex (MHC) class I molecule, creating a neo-antigenic composite that triggers the rapid recruitment, clonotypic TCR recognition, and massive clonal expansion of drug-specific CD8+ cytotoxic T lymphocytes. Activated CD8+ T cells swiftly home to epidermal matrices, orchestrating a profound cytotoxic degranulation cascade and a soluble factor cytokine storm (primarily mediated by IFN-γ and TNF-α) [117]. Keratinocyte death and subsequent full-thickness epidermal necrolysis are executed through the synergistic action of perforin—which creates transmembrane pores—and granzyme B, which enters the host cells to activate the Caspase-3/7 apoptotic pathway [116] (Figure 3).

Concurrently, the massive secretion of granulysin, a potent cationic lipophilic membrane-disrupting protein, independently accelerates widespread keratinocyte apoptosis. For clinicians, this destructive loop culminates in the complete loss of cell–cell adhesion, widespread epidermal detachment, and full-thickness skin sloughing, directly linking the carriage of the HLA-B*58:01 risk allele to life-threatening Stevens–Johnson syndrome and toxic epidermal necrolysis (SJS/TEN) [116,118,119,120].

Crucially, the clinical risk threshold for an individual patient is strictly dictated by the geographic and ancestral distribution of this risk allele [121,122]. The highest global frequencies of HLA-B*58:01 are tightly clustered within Southeast Asian populations, surging to 10–20% among Han Chinese, 8–12% among Thais, and approximately 12% in Korean cohorts, particularly those presenting with concomitant chronic kidney disease due to prolonged oxypurinol retention [114,116,121]. In contrast, African Americans and South Asian Indians display an intermediate risk matrix (allele frequencies of 4% and 2–6%, respectively), while European populations exhibit the lowest baseline risk, with a population frequency of less than 1% [116,118].

The genetic architecture of the Russian Federation populations displays striking ethnic heterogeneity and a sharp geospatial gradient [119,120]. While the prevalence of this immune marker within ethnic Russian groups (Caucasians residing in the European part of the RF) is minimal (<1%), mirroring the general European profile, the complication risks increase significantly in population cohorts harboring a prominent ancestral North Asian genetic component [120].

According to consolidated population genetics data, a critically high frequency of the HLA-B*58:01 allele is fixed among the Kalmyks (~10.3%), a level equivalent to the maximum risk zones of Taiwan and Thailand [119]. An elevated, pathogenetic allele frequency is also characteristic of Buryat and Yakut populations, ranging from 5% to 8% [119,120]. This stark disparity establishes a clinical paradox: whereas current American College of Rheumatology (ACR) guidelines recommend preemptive screening solely for high-risk ancestries (Han Chinese, Thai, African American), extrapolating these standards as a uniform national protocol across the Russian Federation is highly inappropriate [122,123].

Despite a low frequency in the general global population, the odds ratio for an individual carrier remains critically high, ranging from a pooled OR of ~80–120 in multi-ethnic analyses to over 300–600 specifically for SJS/TEN susceptibility within highly homogeneous East Asian cohorts, and comprehensive mapping shows that specific combinations of alternative major histocompatibility alleles (such as HLA-B*58:02, HLA-A*34:02, and HLA-B*35:01) are present in 100% of allopurinol-induced SCAR cases within Caucasian subsets [124]. This underscores the urgent requirement for ethnically oriented, national pharmacogenetic screening algorithms [122]. Preemptive HLA-B*58:01 genotyping must be implemented as a mandatory clinical prerequisite prior to allopurinol initiation for patients belonging to Siberian, and indigenous East/North Asian ethnic groups within the Russian Federation to effectively eliminate life-threatening immunotoxic catastrophes [120,124].

## 7. Monogenic Forms of Hyperuricemia and Systemic Purine Dysregulation

The structural landscape of metabolic purine disorders includes a spectrum of high-penetrance monogenic diseases that trigger severe systemic phenotypes and early-onset gout [125,126,127]. These monogenic variants alter critical enzymatic checkpoints, transforming physiological purine and pyrimidine homeostatic loops into aggressive metabolic cascades [125]. A classic manifestation of X-linked recessive purine dysregulation is HGPRT deficiency, which is structurally driven by pathogenic mutations within the HPRT1 locus at chromosome position Xq26 [125]. HGPRT serves as a fundamental salvage enzyme, recycling free hypoxanthine and guanine back into the nucleotide pool via phosphoribosyl pyrophosphate (PRPP) consumption [126]. Pathogenic variants within the coding sequence disrupt enzyme thermodynamics, yielding a disease spectrum correlated with residual enzymatic kinetics [127,128]. Complete loss-of-function variants resulting in less than 1.5% residual enzymatic activity precipitate Lesch-Nyhan syndrome, characterized by massive uric acid overproduction, early tophi formation, severe extrapyramidal motor deficits (dystonia, choreoathetosis), and pathognomonic self-injurious behavior [128]. Conversely, variants preserving between 1.5% and 8% of functional activity drive Kelley–Seegmiller syndrome (KSS) [127,129]. This residual enzymatic threshold is sufficient to shield the basal ganglia from structural neuro-degeneration but remains highly inadequate for controlling systemic urate levels, causing refractory hyperuricemia, aggressive urolithiasis, and early-onset gouty arthritis during adolescence [129]. Parallel systemic pathogenicity originates from genetic variations in the PRPS1 gene (locus Xq22.3), which encodes the key rate-limiting isoenzyme phosphoribosyl pyrophosphate synthetase 1 [130]. PRPS1 catalyzes the structural conversion of ribose-5-phosphate into PRPP, establishing the baseline substrate driving both de novo purine and pyrimidine nucleotide biosynthesis [129,130]. Mutational profiles in PRPS1 yield a distinct functional dichotomy [131]—loss-of-function variants impair base synthesis, causing severe multi-system neuropathies such as Arts syndrome and X-linked Charcot–Marie–Tooth disease, marked by progressive muscle wasting, optic atrophy, and ataxia [132]. Gain-of-function variants disrupt the structural allosteric binding sites of the enzyme, completely abolishing its sensitivity to purine nucleotide feedback inhibition [131]. This loss of allosteric control causes constitutive hyperactivation of the synthetase, inducing an immediate surge in intracellular PRPP levels, accelerating de novo purine overproduction, and manifesting clinically as refractory hyperuricemia, early-onset gout, and progressive sensorineural hearing loss [131,132]. Beyond constitutional metabolic syndromes, somatic gain-of-function mutations within the PRPS1 locus serve as a driving oncogenic and metabolic mechanism regulating chemotherapy resistance and pediatric acute lymphoblastic leukemia (ALL) relapses [133]. During standard maintenance chemotherapy with thiopurines (e.g., 6-mercaptopurine, 6-MP), selective evolutionary pressure triggers the rapid expansion of sub-clonal tumor cells harboring somatic PRPS1 mutations [133]. Because the mutated enzyme is immune to feedback inhibition, it drives a continuous hyper-production of endogenous natural purines [133]. Within the cytoplasmic matrix, these excessive endogenous purine pools act through competitive inhibition, blocking the initial enzymatic activation step of 6-MP and preventing its conversion into cytotoxic thiopurine nucleotides [134]. This molecular block strips the chemotherapeutic agent of its cytotoxic efficacy, allowing resistant leukemic clones to escape apoptosis and precipitate aggressive clinical relapse [133,134]. Furthermore, PRPS1 hyperexpression acts as a primary metabolic driver of resistance to platinum-based chemotherapies (such as cisplatin) in advanced solid tumors, including breast cancer [135]. The hyperfunctional enzyme maintains an expanded intracellular nucleotide and ATP pool, providing the extensive building resources necessary for the rapid repair of cisplatin-induced DNA double-strand breaks [134]. Concurrently, elevated PRPS1 kinetics maintain exceptionally high levels of intracellular NADPH and reduced glutathione (GSH), facilitating the direct chemical neutralization and detoxification of platinum agents within the cytosol, thereby blocking mitochondrial apoptosis and driving multi-drug refractory tumor survival [134]. In addition to X-linked disorders, autosomal dominant tubulointerstitial kidney disease (ADTKD) syndromes constitute a key monogenic branch driving adolescent gout and hypouricosuric hyperuricemia [135]. Mutations in the uromodulin gene (UMOD, 16p12.3) underlie ADTKD Type 1, where defective folding of uromodulin triggers endoplasmic reticulum stress in the thick ascending limb of Henle, accelerating progressive interstitial fibrosis and causing juvenile gout due to severely impaired renal urate clearance [135]. Mutations in the renin gene (REN, 1q32.1) drive ADTKD Type 2, characterized by early-onset gout, progressive nephropathy, mild hyperkalemia, and childhood anemia. Furthermore, mutations in the transcription factor HNF1B (locus 17q12) induce ADTKD Type 3, wherein adolescent gout is structurally combined with multiple renal cortical cysts and a specific maturity-onset diabetes of the young (MODY5) [135]. Rare autosomal recessive disorders further expand this purine dysregulation matrix via indirect metabolic shifts [136,137]. In von Gierke disease (Glycogen Storage Disease Type I, driven by mutations in G6PC at 17q21.31 or SLC37A4 at 11q23.3), glucose-6-phosphatase deficiency blocks normal glycogenolysis, routing the accumulated glucose-6-phosphate into the pentose phosphate pathway [137]. This activates de novo purine overproduction while simultaneously inducing a severe intracellular lactic acidosis; the excess lactate directly competes with urate for excretion via the proximal transportosome, suppressing renal clearance and provoking severe adolescent gout [137,138]. Similarly, hereditary fructose intolerance (aldolase B deficiency, ALDOB gene at 9q31.1) triggers an immediate metabolic crisis upon fructose ingestion [138]. The rapid trap of intracellular inorganic phosphate into fructose-1-phosphate de-energizes hepatocytes, depleting ATP and causing a massive degradation of adenosine monophosphate (AMP) into a sharp surge of circulating uric acid [139]. Finally, organic acid accumulations during metabolic crises in medium-chain acyl-CoA dehydrogenase deficiency (MCADD, ACADM gene at 1p31.1), alternative nucleotide degradation during muscle strain in myadenylate deaminase deficiency (MADA, AMPD1 gene at 1p13.2), or massive cell breakdown during acute rhabdomyolysis in late-onset carnitine palmitoyltransferase II deficiency (CPT II, CPT2 gene at 1p32.3) all uniformly suppress transportosome clearance or flood the circulating systemic pool with immense purine loads, complicating these rare monogenic syndromes with aggressive secondary urate nephropathy and systemic gout [140,141,142,143].

## 8. The Chronological Evolution of Pharmacotherapy for Uric Acid Metabolic Disorders

The therapeutic modulation of systemic hyperuricemia and its clinical inflammatory sequelae has undergone a profound transformation since antiquity, transitioning from non-selective phytopharmacological extracts to next-generation targeted biologic constructs and precision genotype-matched pharmacotherapies [144,145,146,147,148]. Historical paradigms were established in the second century AD by Galen, who documented the pathognomonic crystalline architecture of gouty tophi, and subsequently refined by Alexander of Tralles, who utilized the autumn crocus (*Colchicum autumnale*) to halt targeted joint flares [144,145]. This observation laid the foundation for the isolation of colchicine—an alkaloid that disrupts microtubule polymerization dynamics by binding to tubulin heterodimers, thereby arresting neutrophil chemotaxis and suppressing MSU-induced NLRP3 inflammasome activation within macrophages [11,19] (Figure 4).

Modern targeted antihyperuricemic intervention emerged in 1966 with the clinical introduction of allopurinol [155]. Allopurinol is a purine-mimetic compound designed to simulate the structural framework of hypoxanthine [11]. Mechanistically, it functions as a competitive substrate for the molybdenum-containing oxidoreductase xanthine oxidase (XO), which converts allopurinol into its primary metabolite, oxypurinol [11,148] (Figure 4). Oxypurinol acts as a coordinated non-competitive inhibitor that remains trapped within the XO active site, inactivating the molybdenum center and suppressing de novo urate synthesis [149]. Despite its extensive historical baseline, allopurinol monotherapy is severely restricted by its critical immunotoxic profile [113]. As detailed previously, its administration in carriers of the HLA-B*58:01 allele presents an absolute molecular contraindication due to the catastrophic development of T-cell-mediated SCARs [116]. Furthermore, its clinical efficacy is routinely compromised by function-altering transportosome mutations; hyperexpression or hyperfunction of the apical/basolateral reabsorption machinery (SLC2A9, SLC22A12) often negates the reduction in synthesis, driving secondary allopurinol resistance and necessitating high-dose titration protocols [85,92].To overcome the architectural liabilities of purine-mimetic agents, the Modern Era introduced high-potency non-purine selective XO inhibitors, led by febuxostat (2008) and topiroxostat (2013) [147,149]. Febuxostat possesses a distinct chemical framework that utilizes non-purine heterocyclic structures to establish comprehensive, high-affinity allosteric occlusion of the XO substrate channel, sealing both the oxidized and reduced enzyme states without mimicking endogenous bases [149]. Clinical efficacy trials confirm that febuxostat provides a significantly superior urate-lowering response in patients presenting with established allopurinol resistance [148,149]. Interestingly, the cellular pharmacokinetics of febuxostat are directly modulated by ABCG2 (BCRP) gene polymorphisms [149,154,155,157,160,161]. Because ABCG2 functions as an active efflux transporter for various medications, loss-of-function variants (such as the p.Q141K variant) delay hepatic and intestinal clearance of febuxostat, paradoxically extending its systemic half-life and accentuating its therapeutic potential, though simultaneously requiring vigilant monitoring for cardiovascular safety axes in specific high-risk cohorts [162]. Simultaneously, the therapeutic spectrum evolved toward the specific selective blockade of the reabsorption machinery [149]. Mid-20th-century classic uricosurics, such as probenecid (1950) and sulfinpyrazone (1959), operated via non-selective organic anion transport blockade, which suffered from rapid off-target interactions, severe drug–drug interference, and high immunogenicity [149]. The emergence of targeted URAT1 blockers, including lesinurad (2015) and dotinurad (2020), marked a definitive pivot toward molecular precision [148,149]. Dotinurad exhibits a high selectivity index for URAT1 over OAT1/OAT3 and ABCG2, meaning it does not inhibit secretory pathways at clinically relevant exposure levels, minimizing drug–drug interactions and nephrotoxic risks [83,149]. Pharmacogenomic fine-mapping indicates that SURIs display maximal utility in “underexcretor” cohorts displaying gain-of-function SLC2A9 risk alleles, effectively restoring fractional urate excretion by re-establishing vector equilibrium [83,163]. For patients presenting with chronic, advanced, or tophaceous gout completely refractory to standard XO inhibitors and SURIs, therapeutic strategies shift toward biological enzyme replacement via pegloticase (2010)—a recombinant mammalian uricase conjugated to monomethoxy polyethylene glycol (PEG) [147,164,165]. Pegloticase circumvents the ancient Miocene hominoid UOX gene pseudogenization by directly mediating therapeutic uricolysis, turning insoluble urate aggregates into labile allantoin [165]. However, the long-term clinical utility of pegloticase is restricted by its pronounced immunogenicity [165,166]. A major cohort of treated individuals, designated as “fast responders,” exhibit rapid treatment failure driven by the synthesis of high-titer anti-drug antibodies (ADAs) directed specifically against the PEG immunogenic shell [166,167]. Immunogenetic studies demonstrate a strong association between specific major histocompatibility complex (HLA) class II haplotypes and the intensity of pegloticase antigen presentation to CD4^+^ helper T cells, accelerating ADA production [167]. Pre-existing anti-PEG antibodies, fixed in a portion of the modern population due to prior non-medical exposure via cosmetics and processed food vectors, further trigger a secondary-type, immediate clearing immune response that eliminates the enzyme from circulation within 2–4 infusions [166,167]. To control the hyper-inflammatory downstream loops triggered by persistent crystal deposition when ULT is restricted, biological cytokine shields have been introduced [19]. Canakinumab (approved by the EMA in 2013 and by the US FDA in 2023) is a high-affinity human monoclonal IgG1 antibody designed to selectively target and neutralize interleukin-1 beta (IL-1β) [19]. By blocking IL-1β binding to cell-surface IL-1 receptors, canakinumab effectively isolates downstream vascular and articular tissue matrices from the massive inflammatory cascade triggered by macro-macrophage NLRP3 inflammasome assembly, providing systemic protection against genetic flares without disrupting baseline urate transportosome dynamics [19,153]. Future perspectives (2026–2030) are firmly defined by the emergence of next-generation, ultra-high-potency small-molecule SURIs currently undergoing advanced Phase III clinical registries, including pozdaftinurad (AR882), ruzinurad, and epaminurad [151,153]. Pozdaftinurad displays unique thermodynamic properties that allow it to completely overcome genetically determined resistance patterns fixed by complex co-carriage of ABCG2 (p.Q141K) and SLC2A9 (rs16890979) risk variants [151,168,169,170,171,172,173,174]. By utilizing an optimized, non-interfering spatial conformation, these next-generation agents stabilize the transportosome macromolecular complex, promising ideal safety metrics, preventing excessive acute uricosuric tubular crystallization, and driving the definitive implementation of the clinical “transportosome genetic passport” [151,169,173,175,176,177,178].

## 9. Evolutionary Aspects of Hyperuricemia and the Molecular Inactivation of the *UOX* Locus

The evolutionary history of the hominoid lineage is fundamentally defined by a profound metabolic reconfiguration: the structural silencing of the *UOX* gene, which encodes the peroxisomal enzyme urate oxidase (uricase) [13,14,179] (Figure 5). While the vast majority of non-primate terrestrial mammals maintain circulating urate levels within a strict, sub-millimolar physiological window via hepatic uricolysis, humans and higher primates lack functional uricase activity entirely [11,12,179]. Consequently, systemic concentration dynamics shift dramatically; the non-degradable end product of purine catabolism in humans is free uric acid, which exhibits an exceptionally low solubility coefficient and prone to crystalline phase transitions [12,15,180].

Phylogenetic mapping and comparative genomics reveal that the loss of uricase function was not an instantaneous, single-nucleotide accident, but a multi-step, sequential process of pseudogenization fixed during the Miocene epoch, approximately 15 to 20 million years ago [14]. Ancestral hominoids underwent at least three independent genetic disruptions within the *UOX* locus [64]. The foundational event was a highly penetrant nonsense mutation within exon 2, converting a codon for arginine into a premature stop codon (Arg33Ter), which is universally conserved across humans, chimpanzees, gorillas, and orangutans [64,179]. While the p.Arg33Ter mutation is universally conserved across humans, chimpanzees, gorillas, and orangutans, separate evolutionary disruptions—such as distinct exon 5 insertions and exon 3 deletions—were fixed independently within the gibbon (Hylobatidae) lineage, demonstrating an intense, parallel evolutionary pressure targeting the active silencing of this metabolic pathway [64].

To understand the macro-evolutionary driving forces behind the retention of these disruptive mutations, evolutionary medicine relies on James Neel’s foundational “thrifty genotype hypothesis” [64,179,180]. During the mid-Miocene, tectonic shifts and global cooling drastically altered equatorial habitats, inducing a prolonged transition from stable tropical rain forests to highly seasonal, open woodland ecosystems [180]. Ancestral hominoids faced severe nutritional stress, extended periods of winter starvation, and geographical isolation [181]. In this stressful ecological context, the complete loss of uricase function provided an immediate, profound survival advantage [182] (Figure 5).

At the cellular level, soluble intracellular urate serves as a potent metabolic rheostat that accelerates fructose-mediated lipogenesis and stimulates de novo fatty acid synthesis within hepatocytes [14,182]. When early hominoids consumed wild fruits rich in fructose, the lack of uricase allowed intracellular urate levels to rise rapidly [183]. This transient intracellular surge induces local mitochondrial oxidative stress via the direct inhibition of aconitase-2 and enoyl-CoA hydratase 1, thereby downregulating the citric acid cycle and blocking beta-oxidation pathways [183,184].

Instead of being burned for immediate energy, the dietary carbohydrates were immediately shunted into triglyceride accumulation within the liver and adipose tissue depots [184]. This metabolic adaptation enabled ancestral hominoids to maximize fat storage from scarce seasonal fruits, establishing an energy buffer that mitigated starvation risks during prolonged famines [185,186]. An intriguing, yet unresolved evolutionary question concerns the precise chronological sequence of these multi-genic modifications: whether ancestral transportosome alterations initially drove the elevation of serum uric acid while uricase was still functionally active, or whether the pseudogenization of the UOX locus occurred first, prompting a subsequent compensatory increase in URAT1 urate affinity to adjust to altered circulating pools [13,14]. While solid molecular evidence verifies that both uricase and URAT1 were concurrently adapting over tens of millions of years, the exact ecological triggers that uniquely drove this anthropoid pathway remain a subject of intense debate [14,30]. Although other mammalian species in the mid-Miocene habitats were subjected to identical ecological and dietary pressures, higher primates possessed a unique metabolic and behavioral matrix—characterized by specialized frugivory, high baseline reliance on vitamin C synthesis loss, and complex cognitive expansion—which made the metabolic shunting of fructose into lipid stores selectively advantageous for them, but not necessarily for sympatric non-primate mammals [13,14,30,183]. The exact selective forces favoring this distinct hominoid trajectory warrant a cautious, moderated interpretation of current evolutionary models [183]. Furthermore, the upregulation of circulating urate levels played a critical role in stabilizing blood pressure under low-sodium conditions and provided a vital antioxidant shield that protected the expanding human neocortex from lipid peroxidation, thus supporting the complex cognitive and structural evolution of the hominid brain [186,187,188,189,190].

In the modern anthropocene, however, this thrifty genetic mechanism has decoupled from its original evolutionary utility [191,192,193]. The continuous availability of purine-dense diets, refined sugars, and high-fructose corn syrup forces a constitutional hyperactivation of this Miocene pathway, transforming a key survival adaptation into the primary molecular driver of modern metabolic syndromes, chronic endothelial inflammation, and visceral vascular injuries [182].

## 10. Conclusions and Future Directions

The transition of hyperuricemia and gout from a localized articular pathology to a systemic metabolic conflict represents a direct consequence of the hominoid evolutionary trajectory. The Miocene pseudogenization of the *UOX* locus established a high-baseline serum urate matrix that initially optimized lipid conservation and antioxidant competence under restrictive ecological selection pressures. However, in modern Westernized dietary landscapes, this ancient metabolic optimization induces structural saturation of the renal and intestinal transportosome, transforming a physiological survival strategy into an independent driver of systemic endothelial damage, subclinical inflammation, and accelerated macrovascular remodeling.

Resolving this pathogenetic dilemma in next-generation clinical medicine necessitates a definitive departure from generalized, population-wide guidelines toward a molecularly stratified, ethnically oriented paradigm. Comprehensive mapping of the renal transportosome confirms that single-nucleotide polymorphisms fixed within *SLC22A12* (URAT1), *SLC2A9* (GLUT9), and *ABCG2* (BCRP) determine distinct “underexcretor” and pharmacoresistant phenotypes that rule out a universal therapeutic approach.

The future clinical architecture must incorporate the mandatory introduction of a personalized “transportosome genetic passport.” This diagnostic algorithm will enable clinicians to preemptively screen high-risk indigenous cohorts of East/North Asian ancestry (such as Kalmyks, Buryats, and Yakuts) for the HLA-B*58:01 allele to absolutely prevent life-threatening, allopurinol-induced immunotoxic catastrophes. Concurrently, structural identification of *ABCG2* (p.Q141K) defects will direct early titration switches to high-affinity selective xanthine oxidase inhibitors (febuxostat) or next-generation, high-potency SURIs currently entering Phase III registries, such as pozdaftinurad (AR882), while avoiding statin-induced cumulative muscle toxicities. Ultimately, integrating evolutionary genomics with membrane structural biophysics and population pharmacogenetics will pave the way for true targeted therapy, establishing maximum personalized disease control and protecting hyperuricemic patient populations from multi-system visceral damage.

## Figures and Tables

**Figure 1:**
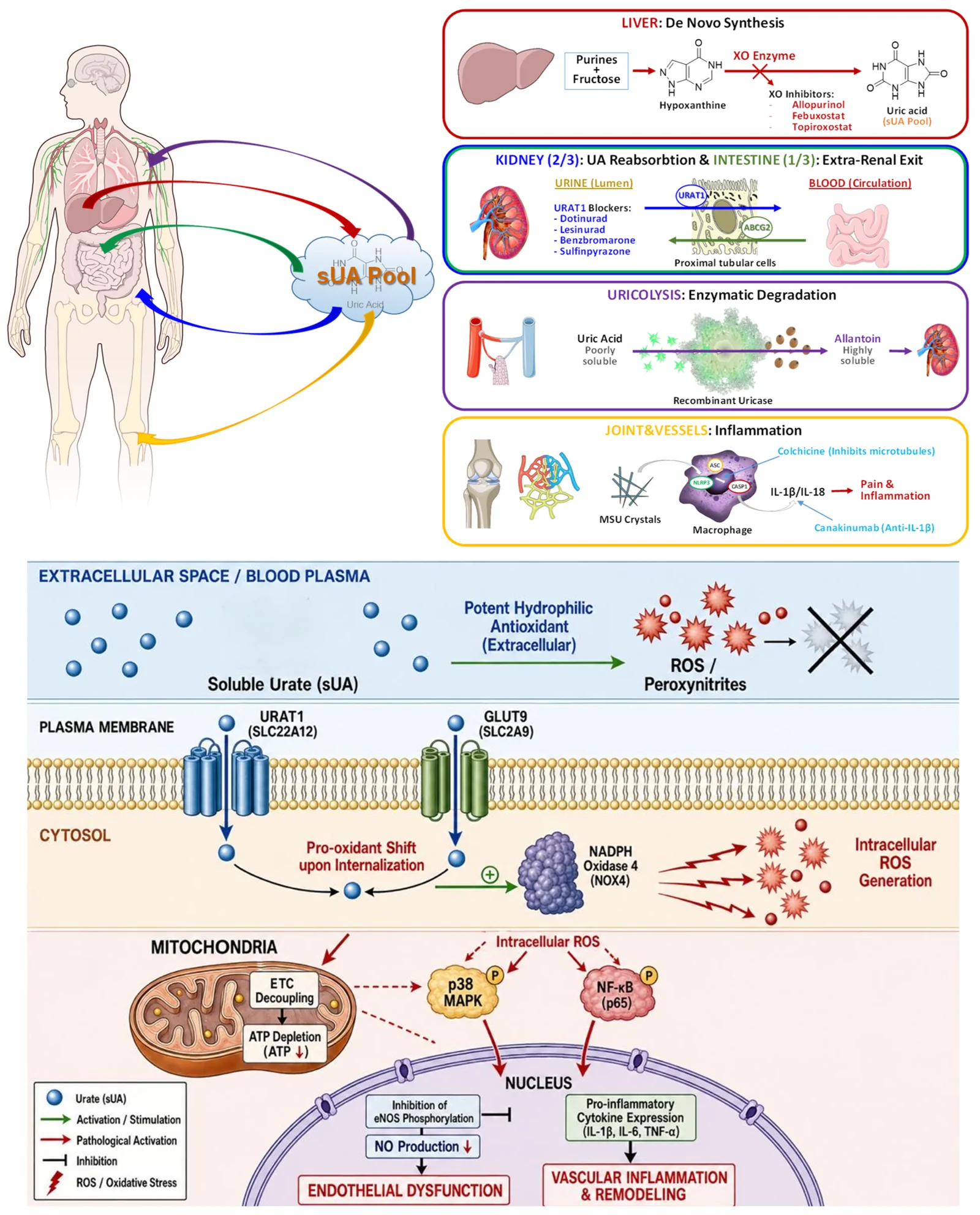
Overview of human uric acid metabolism, elimination pathways, and pharmacological interventions. The schematic diagrams illustrate the comprehensive life cycle of uric acid (UA), integrating its enzymatic de novo synthesis from purines and fructose in the liver via xanthine oxidase (XO), and its subsequent distribution into the serum uric acid (sUA) pool. The principal elimination routes are highlighted, distinguishing between renal excretion (accounting for 70% of total clearance) regulated by the transportosome interactome, and extrarenal intestinal secretion (accounting for 30%). The schematic highlights the dual-site action of the ABCG2 efflux pump, which drives active apical urate rejection into both the intestinal lumen and the renal proximal tubule lumen. The pathway of therapeutic uricolysis shows the recombinant uricase-mediated conversion of poorly soluble urate into highly hydrophilic allantoin. Pathophysiological consequences of supersaturation are depicted via monosodium urate (MSU) crystal deposition, downstream NLRP3 inflammasome activation in macrophages, and the cascade of interleukin-1beta (IL-1β) and interleukin-18 (IL-18) secretion driving localized joint and vascular inflammation. Specific molecular targets for current pharmacological interventions, including XO inhibitors, URAT1 blockers, colchicine, and monoclonal antibodies, are indicated. The p.Q141K variant in ABCG2 characteristically causes a 50% reduction in intrinsic transport function due to nucleotide-binding domain instability, which translates to a striking 29.2% population-attributable risk for developing systemic hyperuricemia.

**Figure 2:**
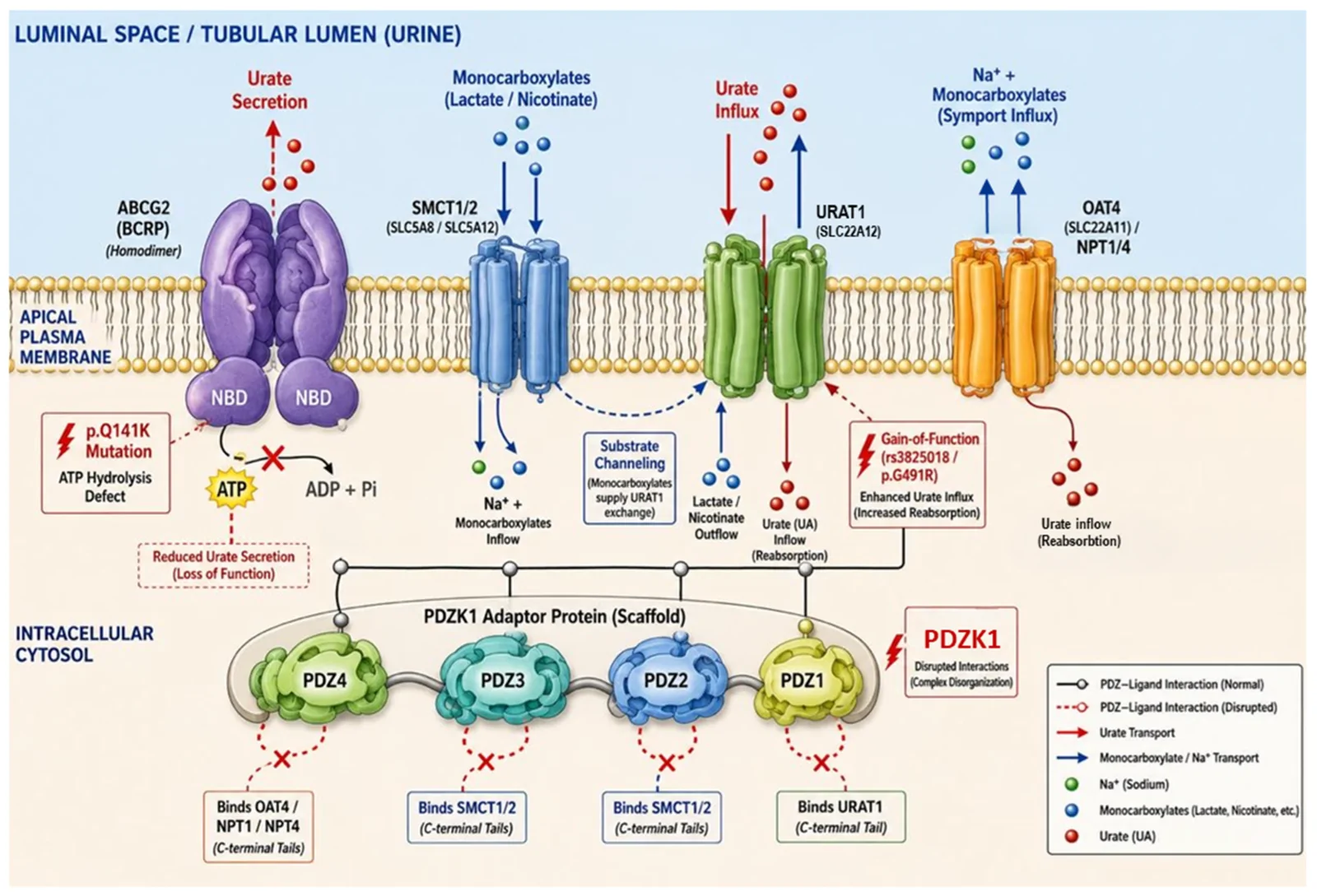
Molecular architecture and transport machinery of the renal proximal tubule epitheliocyte regulating urate homeostasis. The detailed diagram maps the three interconnected functional transport networks structurally organized by the hierarchical PDZK1 scaffold protein on the tubular cell membranes. The reabsorption machinery comprises the selective apical influx transporter URAT1 (encoded by *SLC22A12*) and the basolateral efflux facilitator GLUT9 (encoded by *SLC2A9*), which cooperatively return urate to the systemic circulation. The active secretion pathway is mediated by apical efflux pumps, including MRP4 (*ABCC4*) and ABCG2, working in concert with basolateral organic anion transporters (OAT1/3, encoded by *SLC22A6*/*SLC22A8*) to clear uric acid into the tubule lumen. Energy coupling mechanisms demonstrate how sodium-dependent monocarboxylate transporters SMCT1/2 (*SLC5A8*/*SLC5A12*) establish the prerequisite local anion gradient to power URAT1 antiport kinetics. Molecular binding sites for primary uricosuric and antiuricosuric agents are positioned relative to their specific protein targets. The rs2231142 (p.Q141K) single-nucleotide polymorphism disrupts the ABCG2 ATP-binding cassette domain, leading to a 50% loss of wild-type urate efflux activity. Mechanistically, this high-impact cellular deficit underscores why ABCG2 barrier dysfunction accounts for 29.2% of the population-attributable risk for primary hyperuricemia.

**Figure 3:**
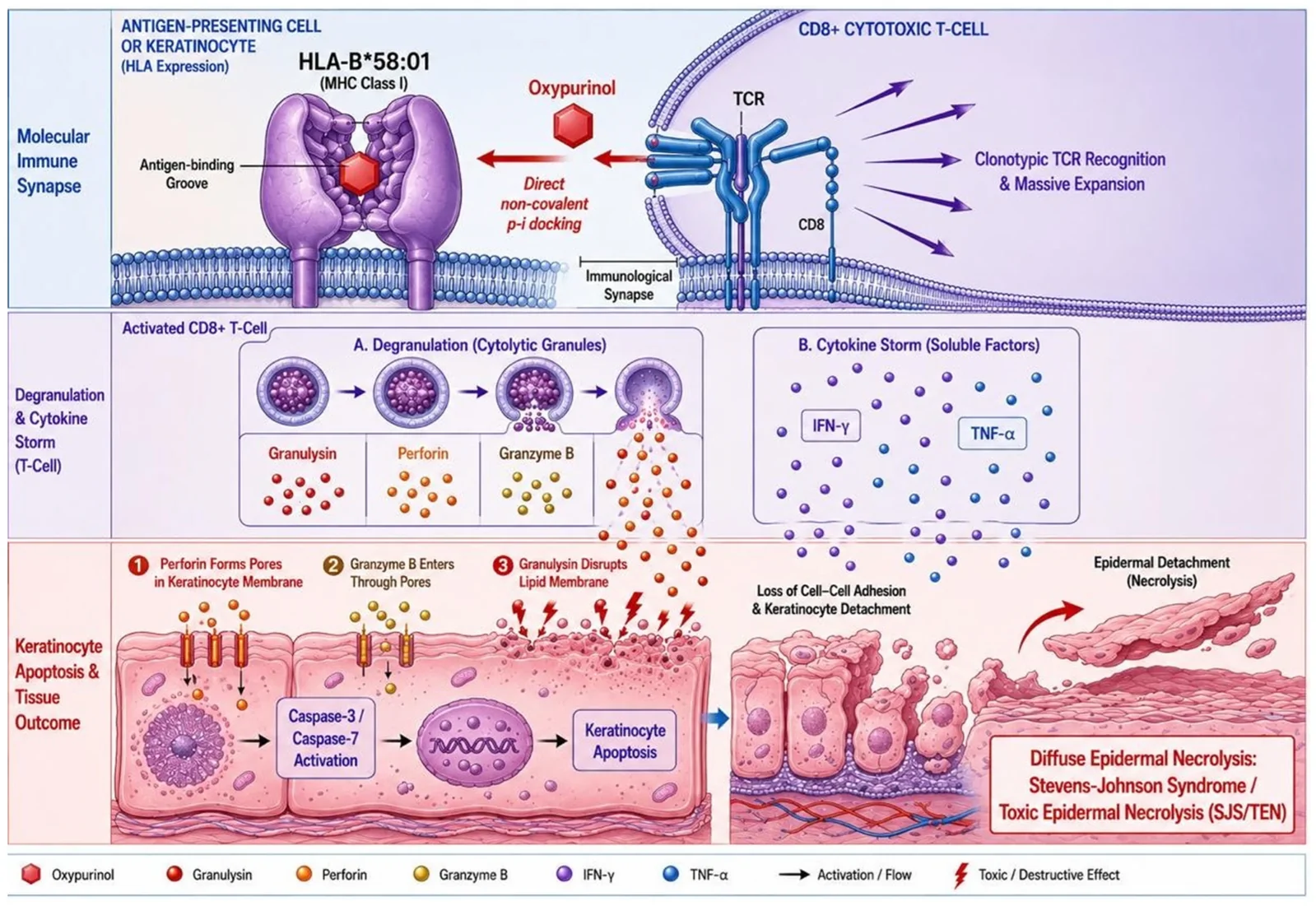
Molecular pathogenesis of allopurinol-induced Stevens–Johnson syndrome and toxic epidermal necrolysis (SJS/TEN). The schematic illustrates the immune-mediated cascade of SCARs triggered by oxypurinol, the active metabolite of allopurinol. (**Top**): According to the p-i concept (direct non-covalent interaction), oxypurinol binds directly to the antigen-binding groove of the HLA-B*58:01 (MHC Class I) molecule on antigen-presenting cells or keratinocytes. This complex promotes clonotypic TCR recognition and robust expansion of CD8+ cytotoxic T-cells. Middle: Activated CD8+ T-cells drive a dual response consisting of degranulation (release of granulysin, perforin, and granzyme B) and a soluble factor cytokine storm (IFN-γ and TNF-α). (**Bottom**): Perforin forms pores in the keratinocyte membrane, enabling granzyme B entry, which subsequent activates Caspase-3/7 and initiates programmed cell death. Granulysin independently disrupts the lipid membrane. Widespread keratinocyte apoptosis culminates in the loss of cell–cell adhesion, epidermal detachment, and full-thickness necrolysis characteristic of SJS/TEN.

**Figure 4:**
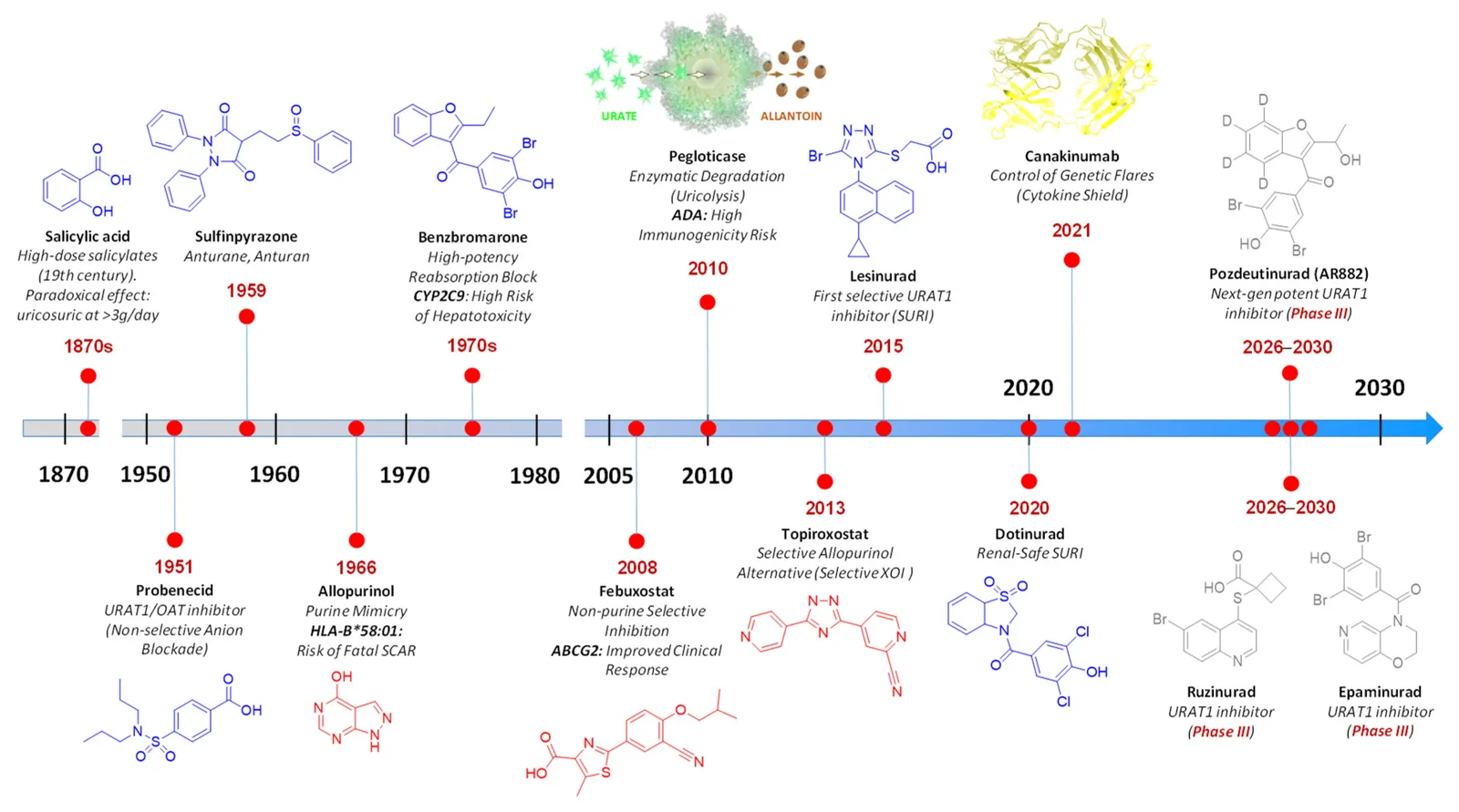
Chronological timeline of pharmacotherapy development for hyperuricemia and gout. The historical evolution of targeted anti-gout medications is traced across four distinct operational eras. The Early Period (1870s–1960s) marks the implementation of high-dose salicylates, the first-generation classic uricosurics probenecid and sulfinpyrazone, and the synthesis of allopurinol as the standard purine-mimetic xanthine oxidase inhibitor. The subsequent developmental period (1970s–1980s) highlights the introduction of high-potency benzbromarone [147,148]. During the early developmental stage (1870s–1960s), agents were introduced that formed the foundation of therapy; however, their current use necessitates rigorous pharmacogenetic monitoring. Allopurinol (1966) remains widely utilized in clinical practice, despite the fact that its administration is associated with a risk of SCARs, including Stevens–Johnson syndrome [33,149]. The Modern Era (2000s–Present) reflects the transition toward high selectivity and biological therapies, including the non-purine selective xanthine oxidase inhibitors febuxostat and topiroxostat, the low-immunogenicity URAT1-selective blockers lesinurad and dotinurad, and the anti-IL-1β monoclonal antibody canakinumab [150,151]. Future Prospects (2026–2030) outline next-generation, ultra-high-potency URAT1 inhibitors currently undergoing advanced Phase III clinical trials, including pozdaftinurad (AR882), ruzinurad, and epaminurad, designed to deliver optimized safety profiles [151,152,153,154,155,156,157,158,159,160,161].

**Figure 5:**
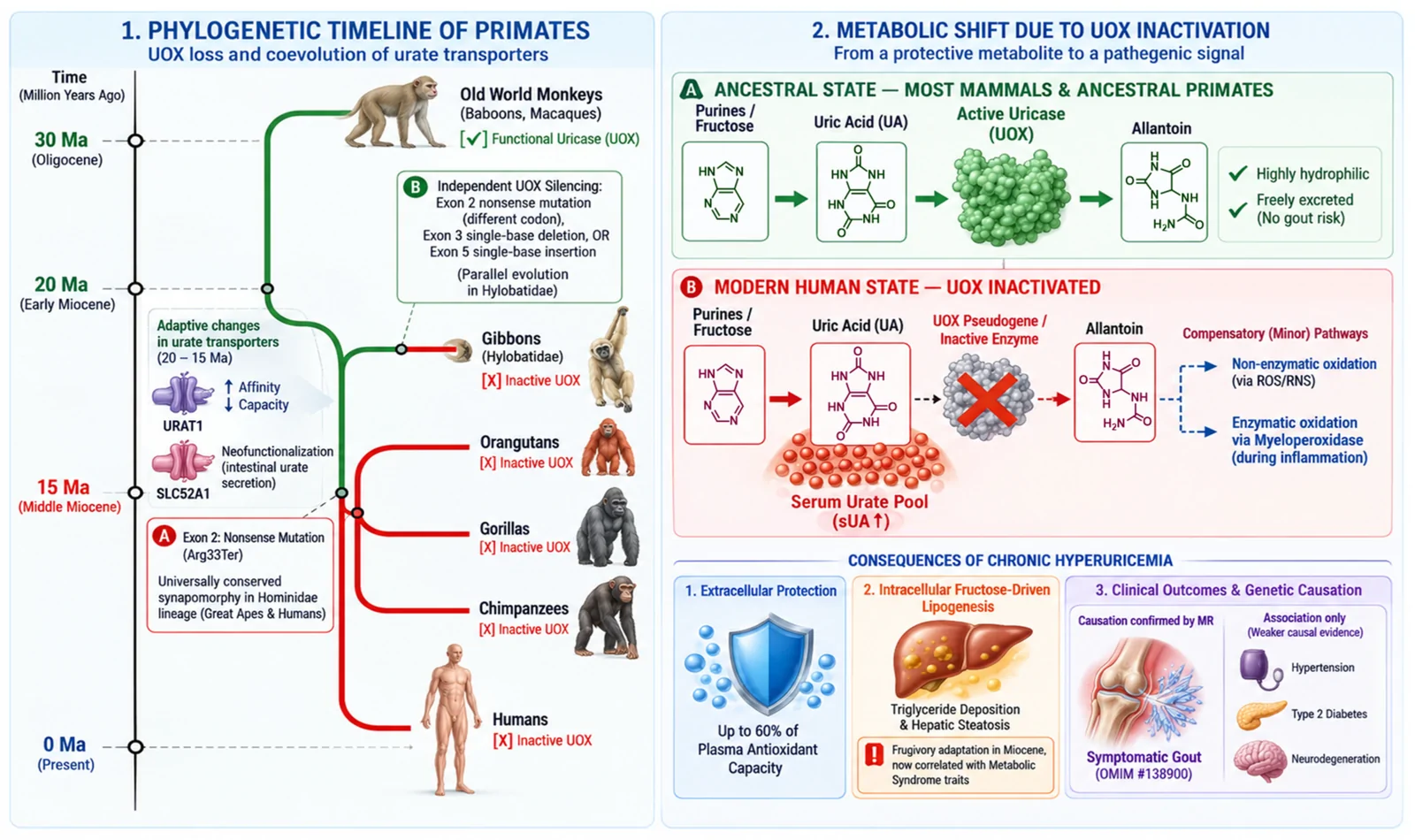
Evolutionary Aspects of the UOX Locus.

## Data Availability

No new data were created or analyzed in this study. Data sharing is not applicable to this article.

## Abbreviations

**Genes and Proteins**	
*ABCG2 (BCRP)*	ATP-Binding Cassette Subfamily G Member 2 (Breast Cancer Resistance Protein)
*ALDOB*	Aldolase B (Fructose-Bisphosphate Aldolase B)
*AMPD1*	Adenosine Monophosphate Deaminase 1
*ASC*	Apoptosis-Associated Speck-Like Protein Containing a CARD
*CPT2*	Carnitine Palmitoyltransferase II
*eNOS*	Endothelial Nitric Oxide Synthase
*G6PC*	Glucose-6-Phosphatase Catalytic Subunit
*GLUT9 (SLC2A9)*	Glucose Transporter Member 9 (Solute Carrier Family 2 Member 9)
*HGPRT (HPRT1)*	Hypoxanthine-Guanine Phosphoribosyltransferase
*HLA*	Human Leukocyte Antigen
*HNF1B*	Hepatocyte Nuclear Factor 1-Beta
*MHC*	Major Histocompatibility Complex
*MRP4 (ABCC4)*	Multidrug Resistance-Associated Protein 4
*NF-κB*	Nuclear Factor Kappa B
*NLRP3*	NOD-Like Receptor Pyrin Domain-Containing 3 (Inflammasome)
*NOX4*	NADPH Oxidase 4
*NPT1/NPT4*	Sodium-Dependent Phosphate Cotransporter 1/4
*OAT1/OAT3/OAT4*	Organic Anion Transporter 1/3/4
*p38 MAPK*	p38 Mitogen-Activated Protein Kinase
*PDZK1*	PDZ Domain-Containing Protein 1
*PRPS1*	Phosphoribosyl Pyrophosphate Synthetase 1
*REN*	Renin
*SLC37A4*	Solute Carrier Family 37 Member 4
*SMCT1/SMCT2*	Sodium-Coupled Monocarboxylate Transporter 1/2
*TCR/TCRs*	T-Cell Receptor/Receptors
*UMOD*	Uromodulin
*URAT1 (SLC22A12)*	Urate Transporter 1 (Solute Carrier Family 22 Member 12)
*UOX*	Urate Oxidase (Uricase gene)
*XO*	Xanthine Oxidase
**Diseases and Clinical Conditions**	
ADTKD	Autosomal Dominant Tubulointerstitial Kidney Disease
AF	Atrial Fibrillation
AKI	Acute Kidney Injury
ALL	Acute Lymphoblastic Leukemia
CKD	Chronic Kidney Disease
DRESS	Drug Reaction with Eosinophilia and Systemic Symptoms
KSS	Kelley–Seegmiller Syndrome
MADA	Myadenylate Deaminase Deficiency
MCADD	Medium-Chain Acyl-CoA Dehydrogenase Deficiency
MODY5	Maturity-Onset Diabetes of the Young, Type 5
SCAR/SCARs	Severe Cutaneous Adverse Reaction/Reactions
SJS	Stevens–Johnson Syndrome
TEN	Toxic Epidermal Necrolysis
**Molecules, Chemicals and Markers**	
6-MP	6-Mercaptopurine
ADA/ADAs	Anti-Drug Antibody/Antibodies
AMP	Adenosine Monophosphate
ATP	Adenosine Triphosphate
DAMP	Danger-Associated Molecular Pattern
DNA	Deoxyribonucleic Acid
GSH	Reduced Glutathione
IFN-γ	Interferon-Gamma
IL-1β/IL-18	Interleukin-1 Beta/Interleukin-18
MSU	Monosodium Urate
NADPH	Nicotinamide Adenine Dinucleotide Phosphate
PEG	Polyethylene Glycol
PRPP	Phosphoribosyl Pyrophosphate
ROS	Reactive Oxygen Species
TNF-α	Tumor Necrosis Factor-Alpha
UA/sUA	Uric Acid/Serum Uric Acid
**Methodology and Pharmacology**	
ACR	American College of Rheumatology
DALYs	Disability-Adjusted Life Years
FEUA	Fractional Excretion of Uric Acid
GWAS	Genome-Wide Association Study
HRT	Hormone Replacement Therapy
LD	Linkage Disequilibrium
MeSH	Medical Subject Headings
OR	Odds Ratio
PRISMA	Preferred Reporting Items for Systematic Reviews and Meta-Analyses
SNP/SNPs	Single-Nucleotide Polymorphism/Polymorphisms
SURI/SURIs	Selective Urate Reabsorption Inhibitor/Inhibitors
ULT	Urate-Lowering Therapy/Therapies
XOI	Xanthine Oxidase Inhibitor
